# Influence of Cooling Lubricants and Structural Parameters on the Tensile Properties of FFF 3D-Printed PLA and PLA/Carbon Fiber Composites

**DOI:** 10.3390/polym17131797

**Published:** 2025-06-27

**Authors:** Aljaž Rogelj, David Liović, Elvis Hozdić, Marina Franulović, Budimir Mijović

**Affiliations:** 1 Faculty of Mechanical Engineering, University of Novo Mesto, Na Loko 2, 8000 Novo Mesto, Slovenia; aljaz.rogelj@fs-unm.si (A.R.); budimir.mijovic@fs-unm.si (B.M.); 2 University of Rijeka, Faculty of Engineering, Vukovarska 58, 51000 Rijeka, Croatia; david.liovic@uniri.hr (D.L.); marina.franulovic@riteh.uniri.hr (M.F.)

**Keywords:** fused filament fabrication (FFF), PLA, PLA+CF, cooling lubricants, tensile properties, infill density, layer thickness, number of perimeters, additive manufacturing (AM), environmental exposure

## Abstract

This study addresses the lack of comprehensive understanding regarding how both structural printing parameters and environmental factors influence the mechanical properties of additively manufactured polymer and composite materials. The main problem stems from insufficient data on the combined effects of infill density, number of perimeters, layer height, and exposure to cooling lubricants on the tensile performance of 3D-printed products, which is crucial for their reliable application in demanding environments. In this research, the influence of four critical parameters—infill density, number of perimeters, layer height, and exposure to cooling lubricants—on the tensile properties of specimens produced by fused filament fabrication (FFF), also known as fused deposition modeling (FDM), from polylactic acid (PLA) and polylactic acid reinforced with carbon fibers (PLA+CF) was investigated. Tensile tests were performed in accordance with ISO 527-2 on specimens printed with honeycomb infill structures under controlled process conditions. The results show that increasing infill density from 40% to 100% led to an approximately 60% increase in tensile strength for both PLA (from 30.75 MPa to 49.11 MPa) and PLA reinforced with carbon fibers (PLA+CF; from 17.75 MPa to 28.72 MPa). Similarly, increasing the number of perimeters from 1 to 3 resulted in a 51% improvement in tensile strength for PLA and 50% for PLA+CF. Reducing layer height from 0.40 mm to 0.20 mm improved tensile strength by 5.4% for PLA and 3.1% for PLA+CF, with more pronounced gains in stiffness observed in the composite material. Exposure to cooling lubricants led to mechanical degradation: after 30 days, PLA exhibited a 15.2% decrease in tensile strength and a 3.4% reduction in Young’s modulus, while PLA+CF showed an 18.6% decrease in strength and a 19.5% drop in modulus. These findings underscore the significant impact of both structural printing parameters and environmental exposure on tailoring the mechanical properties of FFF-printed materials, particularly when comparing unfilled PLA with carbon fiber-reinforced PLA.

## 1. Introduction

Additive Manufacturing (AM), widely referred to as 3D printing, has profoundly transformed the fabrication of polymeric materials, particularly in sectors where rapid prototyping, product individualization, and superior functional performance are critical [1,2,3,4,5,6,7]. Among the spectrum of AM technologies, Fused Filament Fabrication (FFF), also known as Fused Deposition Modeling (FDM) [8,9,10,11,12] has emerged as a leading technique owing to its operational simplicity, cost-efficiency, and compatibility with various thermoplastic polymers [13,14,15,16,17]. Notably, Polylactic Acid (PLA) and its carbon fiber-reinforced composite (PLA+CF) have garnered significant attention due to their capacity to deliver lightweight structures with enhanced mechanical strength, rendering them particularly suitable for engineering applications that demand both structural integrity and reduced mass [18,19,20].

These materials are increasingly employed in both prototyping and end-use components within manufacturing environments, where exposure to industrial fluids, lubricants, or chemically aggressive substances is common.

PLA is favored in FFF due to its environmental compatibility, low cost, and excellent printability [17,21]. It consistently delivers high dimensional accuracy and stable layer deposition. While it has lower tensile strength compared to engineering-grade thermoplastics [13,22], its stiffness and rigidity contribute significantly to maintaining structural integrity. The mechanical performance of PLA—including tensile, flexural, and impact resistance—has been widely reported [17,23,24,25]. Furthermore, its adaptability across different FFF platforms is enhanced by a low melting temperature and reliable impact behavior [26,27].

The incorporation of carbon fibers into polymer matrices has been demonstrated to significantly enhance key mechanical attributes such as tensile strength, rigidity, and thermal resistance [28,29,30]. Despite the broad application of PLA and PLA+CF, limited attention has been paid to their mechanical durability under environmental influences such as temperature, humidity, and chemical exposure [16,18,31,32,33,34]. Contact with industrial lubricants has been shown to provoke microstructural damage, including surface degradation and cracking, which can compromise long-term performance [18]. This study therefore focuses specifically on evaluating the tensile behavior of PLA and PLA+CF after exposure to cooling lubricants—a relevant factor for parts used in fluid-exposed manufacturing contexts.

The impact of infill density on the mechanical and functional performance of components produced via FFF has been the subject of extensive investigation. Empirical studies have demonstrated that changes in infill density significantly influence a range of material properties, including hardness [35], impact toughness [36], fatigue life [37], tribological characteristics [38], and creep resistance [39]. Generally, an increase in infill density leads to improved mechanical strength and dimensional stability, while concurrently reducing surface roughness [40] and water absorption [41]. Furthermore, infill density has been shown to affect dielectric properties [42], overall energy consumption during the printing process [43,44], and the dimensional accuracy of the fabricated geometry [45,46,47], underscoring its critical role in shaping both the structural integrity and environmental responsiveness of FFF-printed parts.

The number of perimeter layers in FFF significantly affects the mechanical properties and reliability of 3D-printed parts. Increasing the number of perimeters enhances tensile strength, interlayer adhesion, and resistance to crack propagation by providing a thicker, more robust outer shell. This leads to improved fracture toughness and surface quality, while also reducing the likelihood of delamination under load. However, using excessive perimeter layers can increase material consumption and print time without proportional gains in strength. Therefore, optimizing the number of perimeters is essential for balancing mechanical performance and production efficiency, and should be considered alongside other parameters such as infill density and layer height [48,49,50]. The number of perimeters layers significantly influences the strength, and increasing them directly leads to an increase in mechanical strength [51]. In the study [52], the authors investigated the influence of air gap between layers and the number of perimeter contours on the flexural strength of polycarbonate FDM prototypes. Using a three-point bending test, they demonstrated that a sparser internal structure can effectively reduce both the mass and printing time of the components while maintaining satisfactory mechanical performance.

Layer thickness, defined as the vertical height of each deposited layer during the FFF process, also significantly affects part quality and strength. Its value depends on nozzle diameter and material characteristics, with reinforced composites posing challenges for extremely fine layer heights (e.g., <0.06 mm) due to flow limitations. Recommendations suggest keeping the layer height below 80% of the nozzle diameter, with an optimal range around 50% [53]. This parameter influences mechanical strength, surface roughness, dimensional precision, and build time [54,55,56,57] making it essential for balancing quality and productivity.

Although previous studies by Hozdić and Hozdić [18] and Hozdić and Hasanagić [31] have examined the effects of individual printing parameters and environmental exposure on the mechanical properties of FFF-printed PLA and PLA+CF, the present study introduces several important novelties. In contrast to earlier work, this research systematically investigates the combined and synergistic effects of infill density, number of perimeters, layer thickness, and prolonged cooling lubricant exposure on tensile properties, utilizing a broader experimental matrix and standardized testing conditions. By directly comparing unfilled and composite PLA materials under identical process parameters and exposure protocols, this study provides a more comprehensive understanding of how both structural and environmental factors influence mechanical performance. Thus, the present work extends and complements previous findings while addressing existing gaps in the literature.

The present study aims to provide a comprehensive assessment of the combined effects of cooling lubricant exposure and selected structural parameters—namely infill density, number of perimeters, and layer thickness—on the tensile properties of FFF 3D-printed PLA and PLA+CF specimens. The overarching objective of this research is to evaluate how lubricant exposure and variations in printing parameters affect key mechanical responses, including ultimate tensile strength, Young’s modulus, and fracture strain. A particular emphasis is placed on comparing the behavior of unfilled PLA with PLA+CF, considering the potential for reinforcing fibers to alter the material’s sensitivity to environmental degradation.

To guide the investigation, several working hypotheses have been formulated. Building upon previous research described in Refs. [18,31], we hypothesize that cooling lubricant exposure reduces tensile strength and ductility in both materials. This effect is expected to be more severe in PLA+CF, owing to its greater stiffness and potential interfacial weakening. Furthermore, higher infill density, more perimeters, and lower layer thickness are anticipated to improve tensile properties. We also expect synergistic effects between structural and environmental factors, whereby the internal geometry modulates sensitivity to lubricant exposure.

This study therefore aims to address a critical gap in the literature by systematically evaluating how the combined influence of structural printing settings and cooling lubricants shapes the tensile behavior of FFF-printed PLA and PLA+CF. By analyzing key mechanical indicators under varied parameter combinations, the research seeks to identify optimal configurations for improved performance and environmental resilience. Importantly, the findings are expected to contribute to more informed material selection and process parameter optimization in industrial additive manufacturing settings, particularly in applications where printed components are routinely exposed to lubricants. Ultimately, the findings are expected to support the reliable deployment of PLA-based composites in real-world manufacturing environments where exposure to lubricants is common, offering new insights for process optimization and material selection in additive manufacturing.

## 2. Materials and Methods

### 2.1. Materials

The experimental investigation employed two thermoplastic filaments: unfilled Polylactic Acid (PLA) and carbon fiber-reinforced PLA (PLA+CF). Both materials were supplied in 1.75 mm diameter spools by [Zhejiang FlashForge 3D Technology Co., Ltd., Zhejiang, China] [58]. The PLA+CF filament consisted of a PLA matrix embedded with short carbon fibers, with a nominal fiber content of approximately 10%.

To reduce the influence of moisture absorption on extrusion stability and interlayer bonding, all filament spools were preconditioned prior to printing. According to manufacturer recommendations [58], PLA filaments were dried at 55 °C for 8 h, while PLA+CF filaments underwent drying at 60 °C for 5 h. The drying process was carried out in a laboratory convection oven. After drying, the materials were stored at 40 °C and ≤20% relative humidity in sealed containers with desiccants to prevent reabsorption of ambient moisture during the course of the experiments.

Material datasheets, including materials specifications and mechanical parameters provided by the manufacturers, are summarized in Table 1 and Table 2.

The decision to use PLA and PLA+CF was based on their wide availability, favorable processing characteristics in FFF systems, and differing mechanical profiles—especially in terms of stiffness, strength, and environmental sensitivity. Their comparative evaluation under lubricant exposure enables a more nuanced understanding of how reinforcement influences material degradation and tensile behavior in additive manufacturing environments.

### 2.2. Specimen Design and Printing Parameters

**Figure 1:**
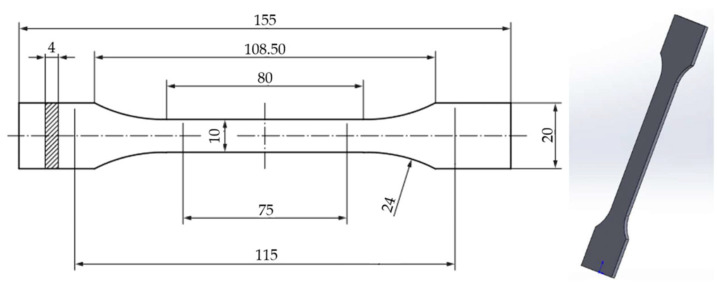
SolidWorks-generated model of standardized tensile specimen geometry (ISO 527-2: 2012) [60,64].

The tensile test specimens were designed using SolidWorks 2020 3D CAD software, following the dimensional and geometrical requirements of the ISO 527-2: 2012 standard [60]. The 3D model (Figure 1) was exported in STL format and subsequently processed using Bambu Studio slicer software (version 01.09.03.50) [63], which was employed to define and adjust all relevant printing parameters prior to fabrication.

All specimens were printed using a Bambu Lab X1 Carbon Combo FFF 3D printer (available online: https://bambulab.com (accessed on 28 July 2024)equipped with a 0.4 mm hardened steel nozzle and an enclosed build chamber. The printer’s precise motion control and stable thermal environment ensured high repeatability. Printing was performed in the flat (XY) orientation to maximize interlayer bonding in the tensile direction.

Printing parameters included both fixed and variable settings. Fixed values such as nozzle diameter (0.4 mm), extrusion temperature (210 °C for PLA, 225 °C for PLA+CF), bed temperature (50 °C), travel speed (100 mm/s), and retraction configuration were set within the ranges recommended by the manufacturer (see Table 3). The base print speed was 60 mm/s, while the first layer speed was limited to 10 mm/s to improve adhesion. Cooling fan settings and retraction (1–2 mm at 30–50 mm/s) followed default slicer profiles for each material.

**Figure 2:**
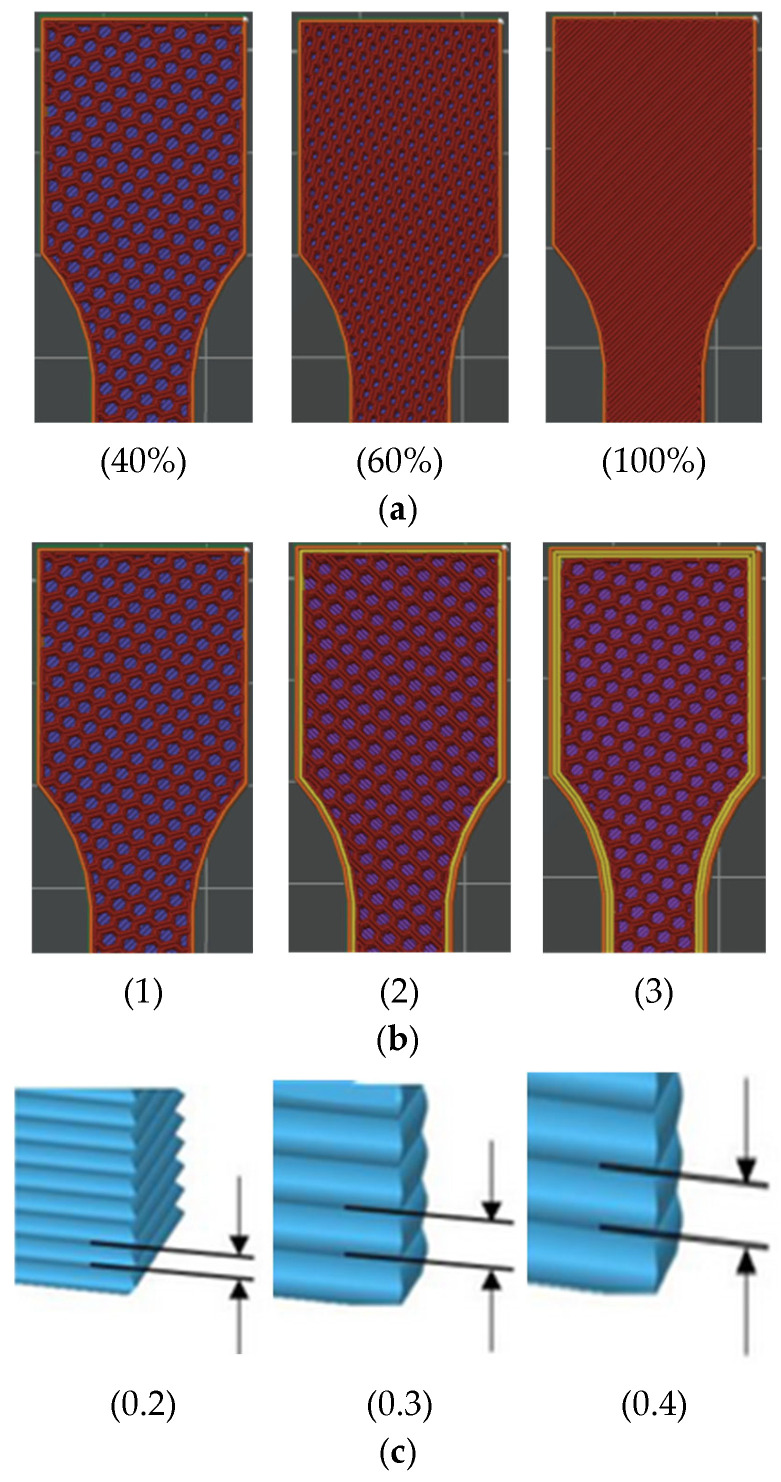
Tensile-test specimens with “*Honeycomb*” and “*Line*” infill pattern: (**a**) 40%, 60%, and 100% infill density, (**b**) 1, 2, and 3 number of perimeters, and (**c**) 0.2-, 0.3-, and 0.4-layer height.

A honeycomb infill pattern was applied for specimens with 40% and 60% infill density, whereas a line infill pattern was used for all specimens printed with 100% infill density (Figure 2). The following process parameters were varied to study their effect on tensile behavior:

- Infill density: 40%, 60%, 100%;
- Number of perimeters: 1, 2, 3;
- Layer height: 0.2 mm, 0.3 mm, 0.4 mm.

**Table 3:** Main printing parameters.

3D Printing Parameter	PLA	PLA+CF
Filament diameter [mm]	1.75	1.75
Infill pattern	*Honeycomb*/*Line*	*Honeycomb*/*Line*
Infill density [%]	40, 60, 100	40, 60, 100
Nozzle diameter [mm]	0.4	0.4
Base print speed [mm/s]	60	60
Travel speed [mm/s]	100	100
First layer maximum [mm/s]	10	10
Top solid layers	1/2/3	1/2/3
Bottom solid layers	1/2/3	1/2/3
Layer height [mm]	0.2/0.3/0.4	0.2/0.3/0.4
First layer height [mm]	0.2/0.3/0.4	0.2/0.3/0.4
Number of perimeters	1/2/3	1/2/3
Extrusion temperature [°C]	210	225
Bed temperature [°C]	50	50

The number of top and bottom solid layers (1, 2, or 3) was coordinated with the selected number of perimeters for each specimen type. Specifically, for each set of printing conditions, the number of top and bottom solid layers matched the number of perimeter shells applied to the specimen (e.g., samples printed with three perimeters were also fabricated with three solid layers at the top and bottom surfaces). This approach was implemented to ensure consistent shell thickness throughout the specimen and to achieve uniform mechanical performance across all tested samples. The corresponding values for each parameter are explicitly presented in Table 3.

The decision to vary infill density, number of perimeters, and layer height was based on their well-documented impact on the mechanical performance of FFF-printed components. Infill density directly influences internal material distribution and load transfer capacity, while the number of perimeters affects the strength and stiffness of the outer shell, which is critical under tensile loading. Layer height governs interlayer adhesion and contributes to both surface quality and structural cohesion. These parameters were selected for systematic variation to capture their individual and interactive effects on tensile strength, Young’s modulus, and strain response, particularly in combination with environmental exposure. Their selection reflects common design considerations in real-world additive manufacturing applications, where mechanical performance must be optimized within process constraints.

Due to the large number of possible combinations when considering all relevant parameters (three levels of infill density, perimeter number, and layer height), a full factorial design would require 27 unique sample types for each material. In this study, a fractional factorial design was adopted to balance comprehensive coverage of parameter space with practical constraints related to time and material resources. Selected combinations were chosen to ensure meaningful comparison of low (40%), intermediate (60%), and high (100%) infill densities, as well as to capture the effects of varying the number of perimeters and layer thicknesses. Certain combinations, such as varying layer height at 40% infill, were not included, as preliminary investigations and a review of the literature suggest that the influence of some parameters is most pronounced at higher infill densities. This focused approach allows for efficient statistical analysis of main effects while optimizing experimental effort. The experimental matrix is summarized in Table 3.

Printing was conducted under controlled environmental conditions at 22 ± 1 °C and relative humidity below 20%, in line with best practices for PLA-based materials to minimize moisture-related variability.

### 2.3. Lubricant Exposure Procedure

To assess the effect of chemical exposure on the mechanical performance of FFF-printed PLA and PLA+CF specimens, an immersion protocol was implemented using a widely applied industrial coolant-lubricant. The selected medium was CIMTECH A31F (UN3082) CIMCOOL Europe B.V., Rotterdam, The Netherlands, a water-soluble synthetic lubricant formulated for metalworking applications. A 7% solution was prepared by diluting the concentrate with distilled water, in accordance with the manufacturer’s specifications.

Specimens were fully immersed in the prepared solution and stored at room temperature (22 ± 1 °C) in sealed, chemically inert containers to prevent evaporation and contamination. The exposure durations were defined as 0 (unexposed), 7, and 30 days, selected to simulate short-term and prolonged environmental contact scenarios representative of real-world industrial conditions. During immersion, specimens were placed horizontally with adequate spacing to ensure uniform exposure on all surfaces.

After the designated exposure period, specimens were removed from the solution and gently rinsed with distilled water to eliminate any residual surface contaminants. They were subsequently dried with lint-free cloths and conditioned in ambient laboratory air (22 ± 1 °C, relative humidity ≤ 20%) for 24 h. No forced heating or mechanical drying was applied to avoid introducing artifacts into the mechanical behavior of the material.

Unexposed specimens (0-day group) served as the control condition for comparative evaluation. All tensile testing was performed under identical environmental and procedural conditions across all exposure groups.

### 2.4. Tensile Testing Procedure

Tensile tests were performed to characterize the mechanical behavior of PLA and PLA+CF specimens under uniaxial loading. All measurements were carried out on a Step LAB electromechanical actuator (STEP Lab Engineering S.r.l., Resana, Italy), equipped with a force sensor of 25 kN. Test control and data acquisition were managed using the manufacturer’s proprietary software suite, which enabled continuous recording of force and displacement throughout the experiment.

The procedure followed the ISO 527-2: 2012 standard for plastic materials [60]. The crosshead displacement rate was set to 5 mm/min, as recommended for the tested specimen geometry. The Epsilontech 3442-010M-050M-ST (South Park, WY, USA) extensometer with the gauge length of 50 mm has been used for strain measurements.

Each specimen was loaded to failure, and the stress–strain data were analyzed to extract the following mechanical parameters: tensile strength, Young’s modulus, and fracture strain. Young’s modulus has been determined according to instructions stated in ISO 527-1 standard, by applying the linear regression (least-squares regression line fit) in the 0.05%<strain<0.25% region.

For each test condition—defined by material type, structural parameter configuration, and lubricant exposure duration—five replicates were analyzed to ensure statistical validity. Raw tensile data were exported and post-processed using a custom-written Python version 3.11 script, which enabled automated extraction of mechanical parameters, curve plotting, and consistency checks across all replicates. Damage criterion has been set to 50% of UTS in post processing phase to plot corresponding stress–strain curves and to calculate fracture strain.

### 2.5. Definition of Specimen Code

To enable systematic analysis and clear identification of the tested samples, a specimen code was generated for each combination of process parameters. The code consists of several elements in the following order:

- The first part denotes the material (e.g., PLA or PLA+CF);
- The first digit indicates infill density (e.g., 4 for 40%, 6 for 60%, 1 for 100%);
- The second digit represents the number of perimeters;
- The third and fourth digits together specify the layer height (e.g., 02 for 0.20 mm, 03 for 0.30 mm, 04 for 0.40 mm);
- The fifth character refers to the infill structure (H for honeycomb, L for line);
- The final digit is the specimen’s serial number within its group (Case).

For example, the code PLA_4202H1 refers to a PLA specimen printed with 40% infill density, 2 perimeters, 0.20 mm layer height, honeycomb infill, and serial number 1.

All specimen codes and corresponding parameter values are listed in Table 3 for full transparency and reproducibility.

## 3. Results

### 3.1. Tensile Behavior of the FFF 3D-Printed PLA Specimens

The tensile behavior of FFF 3D-printed PLA specimens was comprehensively evaluated by analyzing the influence of infill density (Section 3.1.1), the number of perimeters (Section 3.1.2), layer height (Section 3.1.3), and exposure to a cooling lubricant (Section 3.1.4).

#### 3.1.1. The Effect of Infill Density on the Tensile Properties of the FFF 3D-Printed PLA Specimens

To evaluate the influence of infill density on the tensile properties of FFF 3D-printed PLA specimens, three groups were prepared, each representing a distinct infill level (Table 4). Specimen codes were systematically assigned to indicate the relevant printing parameters for traceability.

*Case 1* includes specimens labeled PLA_4302H1 to PLA_4302H5, representing the lowest infill density considered in this study (40% infill density). *Case 2* comprises specimens PLA_6302H1 to PLA_6302H5, fabricated with an intermediate infill density (60% infill density). Finally, *Case 3* includes specimens PLA_1302L1 to PLA_1302L5, printed with the highest infill density (100% infill density). The applied coding system ensured the accurate traceability of each sample with respect to its fabrication parameters. The resulting tensile data—comprising maximum force, tensile strength, fracture strain, and Young’s modulus—served as the basis for quantifying the mechanical response of PLA under uniaxial loading as a function of infill density.

**Figure 3:**
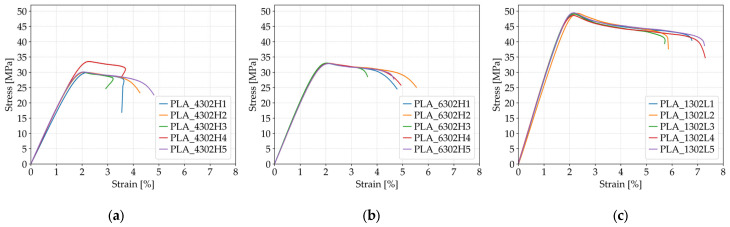
Stress–strain curves of FFF 3D-printed PLA specimens with different infill densities: (**a**) 40% infill (*Case 1*), (**b**) 60% infill (*Case 2*), and (**c**) 100% infill (*Case 3*).

Figure 3 illustrates the tensile strength and fracture strain values for individual FFF 3D-printed PLA specimens produced with varying infill densities. The results highlight the effect of internal structure density on the mechanical performance under uniaxial loading conditions.

The influence of infill density on the tensile strength of FFF 3D-printed PLA specimens with a *Honeycomb* internal structure was found to be substantial. At 40% infill density (*Case 1*), the average tensile strength was measured at 30.75 MPa, increasing to 32.94 MPa at 60% infill (*Case 2*), and reaching a maximum of 49.11 MPa at full infill (*Case 3*). This represents an approximate 60% improvement in tensile strength between the lowest and highest infill configurations. The honeycomb pattern, when employed at higher densities, clearly contributed to enhanced interlayer bonding and a greater volume of material per unit area, which in turn facilitated more uniform stress distribution and reduced the likelihood of crack initiation. In addition to the increase in strength, specimens with 40% infill exhibited the highest standard deviation (1.57 MPa), indicating reduced consistency and higher variability in mechanical performance under lower-density conditions.

Fracture strain results also exhibited a clear upward trend in correlation with increasing infill density. The average fracture strain at 40% infill was recorded at 3.92%, rising to 4.71% at 60% infill, and reaching 6.58% at 100% infill. The *Honeycomb* geometry, when implemented at higher densities, provided more effective stress transmission between layers and a structurally more homogeneous internal matrix, which contributed to improved ductility. The highest fracture strain values, observed in the specimens from *Case 3*, confirm that a fully filled *Honeycomb* structure not only ensures high strength but also offers excellent energy absorption capacity under tensile loading, delaying the onset of failure and allowing for greater deformation without sudden fracture.

**Figure 4:**
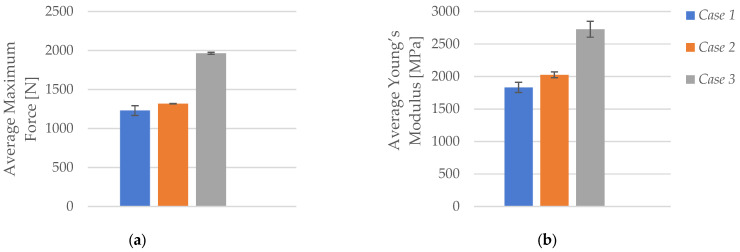
Average mechanical properties of FFF 3D-printed PLA specimens with different infill densities: (**a**) average maximum force, and (**b**) average Young’s modulus.

As illustrated in Figure 4a, the average maximum force increased significantly with higher infill densities. Specimens with 40% infill (*Case 1*) exhibited an average maximum force of 1230.18 N, which rose to 1317.71 N at 60% infill (*Case 2*), and peaked at 1964.62 N for fully infilled specimens (*Case 3*). This represents an approximate 60% enhancement in load-bearing capacity, attributable to the greater material volume and more effective internal stress distribution provided by the denser *Honeycomb* structure.

Figure 4b shows a similar trend for Young’s modulus, reflecting improved stiffness with increasing infill density. The modulus rose from 1832.71 MPa at 40% infill to 2025.72 MPa at 60%, reaching 2727.08 MPa at 100%. The denser *Honeycomb* configuration contributed to reduced void content and enhanced structural cohesion, resulting in greater elastic resistance under uniaxial tensile loading.

#### 3.1.2. Effect of the Number of Perimeters on the Tensile Properties of the FFF 3D-Printed PLA Specimens

To evaluate the effect of the number of perimeters on the tensile properties of FFF 3D-printed PLA specimens, three sets of samples were produced using a constant infill density of 60% and a *Honeycomb* internal structure, while varying the number of perimeters (outer walls). As presented in Table 5, *Case 4* includes specimens PLA_6102H1 to PLA_6102H5, printed with one perimeter. *Case 5* consists of specimens PLA_6202H1 to PLA_6202H5, fabricated with two perimeters, and *Case 6* comprises specimens PLA_6302H1 to PLA_6302H5, manufactured with three perimeters. The resulting data for tensile strength, fracture strain, maximum force, and Young’s modulus were analyzed to assess how the increasing number of perimeters influences the mechanical performance of PLA components under uniaxial tensile loading.

**Figure 5:**
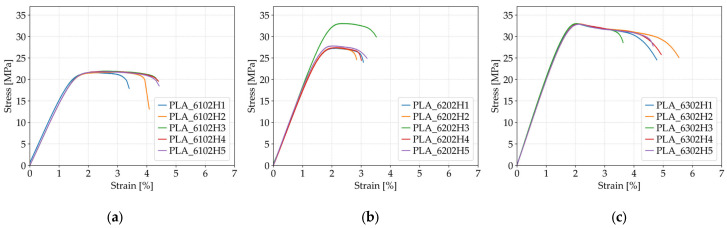
Tensile stress–strain curves of FFF 3D-printed PLA specimens with varying numbers of perimeters: (**a**) one perimeter (*Case 4*), (**b**) two perimeters (*Case 5*), and (**c**) three perimeters (*Case 6*).

Figure 5 illustrates the tensile behavior of PLA specimens fabricated with a constant infill density of 60% and a *Honeycomb* internal structure, while varying the number of perimeters. Increased perimeter count contributed to improved tensile strength and fracture strain, as reflected in the progression from subfigure (a) to (c).

The influence of the number of perimeters on the tensile strength of FFF 3D-printed PLA specimens, as summarized in Table 5, reveals a distinct increasing trend with the addition of outer shell layers. Specimens printed with one perimeter (*Case 4*) exhibited an average tensile strength of 21.79 MPa. When the number of perimeters was increased to two (*Case 5*), the strength improved to 28.59 MPa and further increased to 32.94 MPa with three perimeters (*Case 6*). This represents a total increase of over 50% in tensile strength from *Case 4* to *Case 6*. The improved performance can be attributed to the greater contribution of the perimeter walls in bearing the applied load, especially in the early stages of deformation. Additional perimeters provide a stronger and more continuous outer structure, reducing stress concentrations and improving the overall resistance to crack propagation under tensile loading.

A similar trend is observed in the fracture strain, with specimens exhibiting increased ductility as the number of perimeters rises. The average fracture strain for *Case 4* was 4.13%, which decreased slightly to 3.13% for *Case 5*, but then significantly increased to 4.71% in *Case 6*. Although the intermediate case (two perimeters) showed a slight reduction in fracture strain compared to *Case 4*, the overall data indicate that specimens with three perimeters possessed both high strength and considerable deformation capacity. This is likely due to the improved interlayer bonding and enhanced structural integrity resulting from the thicker shell configuration. The enhanced fracture strain performance in *Case 6* confirms that increasing the number of perimeters not only contributes to strength but also supports better energy dissipation and delayed failure during tensile loading.

**Figure 6:**
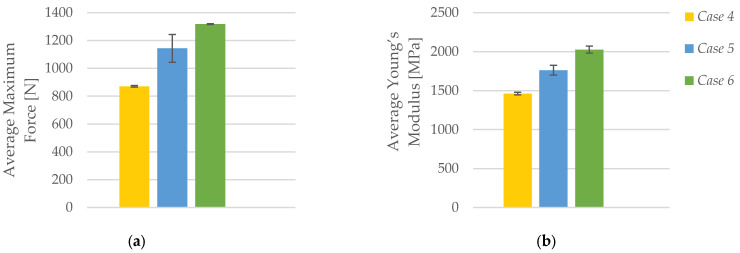
Effect of the number of perimeters on the average mechanical properties of FFF 3D-printed PLA specimens: (**a**) average maximum force, and (**b**) average Young’s modulus.

As shown in Figure 6a, the average maximum force recorded during tensile testing increased notably with a higher number of perimeters in the FFF 3D-printed PLA specimens. Specimens with one perimeter (*Case 4*) exhibited an average peak force of 871.79 N. This value rose to 1143.89 N in specimens with two perimeters (*Case 5*), and further increased to 1317.71 N for those with three perimeters (*Case 6*). The progressive enhancement in maximum load capacity—approximately a 51% increase from *Case 4* to *Case 6*—confirms the significant structural contribution of additional perimeter layers. With each added perimeter, the outer shell provides improved continuity and reinforcement, allowing the specimens to endure greater loads before failure. These results demonstrate that perimeter configuration is a critical design parameter for enhancing load-bearing capacity in printed PLA components.

The trend observed for the average Young’s modulus closely parallels that of the average maximum force, as illustrated in Figure 6b. The modulus increased from 1461.99 MPa in *Case 4* (one perimeter) to 1761.87 MPa in *Case 5* (two perimeters) and reached 2025.72 MPa in *Case 6* (three perimeters). This reflects a total increase of over 38% in stiffness across the tested range. The improved elastic response is attributed to the increased shell thickness, which reduces localized deformation and provides greater structural resistance to tensile elongation. The addition of perimeters not only enhances the material’s strength but also contributes to its rigidity, making it more suitable for load-bearing applications where dimensional stability under stress is essential. These findings reinforce the mechanical benefits of increasing the number of perimeters in FFF-printed PLA structures.

#### 3.1.3. Effect of Layer Height on the Tensile Properties of the FFF 3D-Printed PLA Specimens

To investigate the effect of layer height on the tensile properties of FFF 3D-printed PLA specimens, three sets of samples were fabricated using identical infill density (60%), two perimeters, and a *Honeycomb* infill pattern, while varying the layer height. As shown in Table 6, each group of specimens was assigned a unique specimen code reflecting the printing parameters. *Case 5* includes specimens PLA_6202H1 to PLA_6202H5, printed with a layer height of 0.20 mm. *Case 7* comprises specimens PLA_6203H1 to PLA_6203H5, fabricated with a layer height of 0.30 mm, while *Case 8* includes specimens PLA_6204H1 to PLA_6204H5, manufactured with a layer height of 0.40 mm. This classification ensures the consistent comparison of mechanical performance as a function of layer thickness. The tensile strength, fracture strain, maximum force, and Young’s modulus were analyzed to quantify the impact of increasing layer height on the mechanical response of PLA materials.

**Figure 7:**
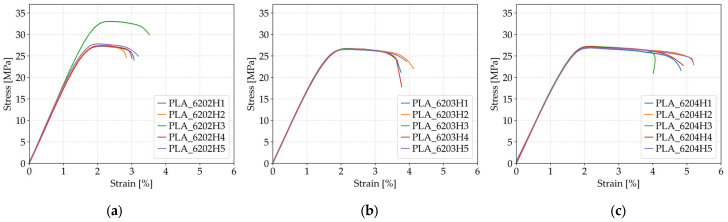
The stress–strain behavior of the specimens with varying layer heights: (**a**) corresponds to specimens printed with a 0.20 mm layer height (*Case 5*), (**b**) to specimens with a 0.30 mm layer height (*Case 7*), and (**c**) to specimens with a 0.40 mm layer height (*Case 8*).

The stress–strain behavior of the specimens with varying layer heights is illustrated in Figure 7.

The influence of layer height on the tensile strength of FFF 3D-printed PLA specimens is evident from the comparative data in Table 6 and Figure 7. Specimens printed with a 0.20 mm layer height (*Case 5*) exhibited the highest average tensile strength at 28.59 MPa. When the layer height was increased to 0.30 mm (*Case 7*), the tensile strength decreased to 26.65 MPa and remained similar at 27.13 MPa for specimens printed with a 0.40 mm layer height (*Case 8*). Although the difference between 0.30 mm and 0.40 mm was minimal, the decrease from the finest to coarsest resolution (0.20 mm to 0.30 mm) was more notable. This decline in strength can be attributed to the reduced interlayer contact area and weaker bonding between adjacent layers at higher layer heights, which leads to a greater likelihood of failure initiation along layer boundaries. These results confirm that finer layer resolution promotes better interlayer adhesion, contributing to enhanced load resistance under tensile stress.

In contrast, the results for fracture strain reveal an increasing trend with greater layer height. The lowest average fracture strain was observed in *Case 5* (0.20 mm), at 3.13%, while *Case 7* (0.30 mm) exhibited an increased fracture strain of 3.85%, and *Case 8* (0.40 mm) reached the highest value at 4.83%. These findings suggest that specimens printed with thicker layers possess greater ductility, allowing for higher elongation before fracture. The enhanced fracture strain values at higher layer heights may be due to increased layer thickness absorbing more deformation energy and delaying crack propagation, despite slightly weaker interfacial strength. Therefore, while finer layers yield higher tensile strength, coarser layers may provide more favorable strain behavior, highlighting the inherent trade-off between strength and ductility when selecting layer height in FFF-printed PLA components.

**Figure 8:**
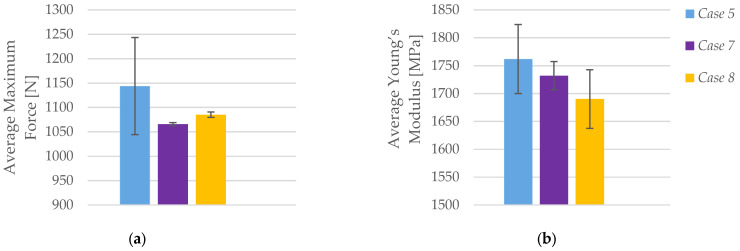
Effect of layer height on the average mechanical properties of FFF 3D-printed PLA specimens: (**a**) average maximum force, and (**b**) average Young’s modulus.

As shown in Figure 8a, the average maximum force recorded during tensile testing decreases with increasing layer height. Specimens printed with a 0.20 mm layer height (*Case 5*) exhibited the highest average maximum force at 1143.89 N, while those printed with 0.30 mm (*Case 7*) and 0.40 mm (*Case 8*) layer heights showed reduced values of 1065.88 N and 1085.07 N, respectively.

A similar trend can be observed in Figure 8b, which presents the average Young’s modulus for each group. The highest stiffness was measured in *Case 5* (1761.87 MPa), followed by *Case 7* (1732.01 MPa) and *Case 8* (1690.10 MPa). The gradual decrease in modulus with increasing layer height suggests that thicker layers result in slightly less stiff components, likely due to reduced interfacial bonding and increased potential for anisotropic deformation. While the changes in modulus are not drastic, they reflect a consistent pattern in which lower layer heights contribute to higher structural rigidity. These findings underscore the importance of optimizing layer height to balance stiffness, strength, and productivity in FFF 3D printing of PLA.

#### 3.1.4. Effect of Lubricant Exposure on the Tensile Properties of the FFF 3D-Printed PLA Specimens

To investigate the effect of lubricant exposure on the tensile properties of FFF 3D-printed PLA specimens, three sets of samples were prepared using identical printing parameters—60% infill density, *Honeycomb* structure, 0.2 mm layer height, and three perimeters—while varying the duration of immersion in the CIMTECH A31F water-based cooling lubricant. As summarized in Table 7, *Case 6* comprises specimens PLA_6302H1 to PLA_6302H5, which were not exposed to lubricants and serve as the control group. *Case 9* includes specimens PLA_6302H71 to PLA_6302H75, which were exposed to the lubricant for 7 days, while *Case 10* consists of specimens PLA_6302H31 to PLA_6302H35, which were immersed for 30 days.

The comparison between the unexposed and exposed groups provides insight into the environmental sensitivity of PLA materials when used in lubricated operating environments.

**Figure 9:**
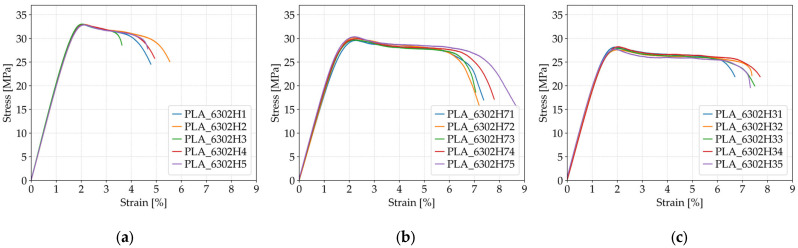
Stress–strain curves of FFF 3D-printed PLA specimens subjected to different durations of lubricant exposure: (**a**) unexposed (*Case 6*), (**b**) exposed for 7 days (*Case 9*), and (**c**) exposed for 30 days (*Case 10*).

As illustrated in Figure 9, the tensile strength of the PLA specimens decreased progressively with longer exposure to the cooling lubricant. The control group (*Case 6*), which was not exposed to the lubricant, exhibited the highest average tensile strength of 32.94 MPa. After 7 days of immersion (*Case 9*), the average tensile strength dropped to 29.96 MPa and further declined to 27.94 MPa following 30 days of exposure (*Case 10*). This corresponds to an overall reduction of approximately 15% between the unexposed and longest-exposed groups. The degradation in tensile strength can be attributed to the chemical interaction between the PLA matrix and the water-based lubricant (CIMTECH A31F), which likely affects the interlayer bonding and polymer chain integrity, leading to reduced load-bearing capacity.

The fracture strain increased with exposure time, as shown in the elongation behavior in Figure 9. The average fracture strain for the unexposed specimens (*Case 6*) was 4.71%, which increased significantly to 7.63% after 7 days (*Case 9*), and remained relatively high at 7.32% after 30 days (*Case 10*). This trend suggests that lubricant exposure induces a plasticizing effect, enhancing the ductility of PLA by enabling greater molecular mobility under tensile loading. However, this gain in ductility comes at the cost of reduced tensile strength, indicating a typical trade-off between strength and flexibility under environmental influence. These findings highlight the susceptibility of PLA to environmental degradation and should be considered in applications where prolonged exposure to lubricants or moisture is expected.

**Figure 10:**
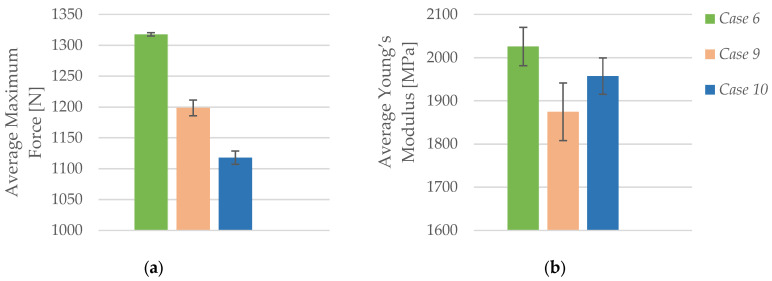
Effect of lubricant exposure duration on the average mechanical properties of FFF 3D-printed PLA specimens: (**a**) average maximum force, and (**b**) average Young’s modulus.

As depicted in Figure 10a, the average maximum force exhibited a clear decreasing trend with increasing lubricant exposure duration. The unexposed specimens (*Case 6*) achieved the highest average force of 1317.71 N, while specimens exposed for 7 days (*Case 9*) showed a reduced average of 1198.57 N, and those exposed for 30 days (*Case 10*) reached only 1117.78 N. This corresponds to a cumulative decline of approximately 15% across the exposure range. The reduction in maximum force suggests that extended immersion in the CIMTECH A31F lubricant weakens the material’s load-bearing capacity, likely due to moisture absorption and the degradation of interfacial adhesion within the layered structure.

A comparable trend is evident in Figure 10b, which illustrates the changes in Young’s modulus. The modulus decreased from 2025.72 MPa in unexposed specimens (*Case 6*) to 1874.71 MPa after 7 days of exposure (*Case 9*), and further to 1957.08 MPa after 30 days (*Case 10*). While a partial recovery in stiffness is observed in *Case 10*, likely due to post-immersion reorganization or partial re-crystallization effects, the overall trend confirms a softening effect resulting from environmental exposure. These findings emphasize that even water-based lubricants can significantly impact the stiffness and load resistance of PLA over time, underlining the importance of environmental resistance in material selection for functional applications.

#### 3.1.5. Summary of PLA Performance Trends

The mechanical performance of FFF 3D-printed PLA specimens was evaluated under four distinct experimental conditions: variation in infill density, number of perimeters, layer height, and duration of lubricant exposure. The results consistently demonstrated that all investigated parameters significantly influence the tensile response of PLA, both in terms of strength and ductility.

An increase in infill density (from 40% to 100%) resulted in substantial improvements in tensile strength and Young’s modulus, with the highest values observed at full infill. This enhancement is attributed to reduced internal voids and improved structural continuity, which enable better load distribution and stress transfer. Similarly, increasing the number of perimeters from one to three led to marked gains in both maximum force and tensile stiffness. The additional shell layers contributed significantly to the structural integrity, especially under axial loading conditions, by reinforcing the outer regions where tensile stresses are highest.

In contrast, increasing the layer height (from 0.20 mm to 0.40 mm) had an inverse effect on mechanical performance. Finer layer resolutions resulted in higher tensile strength and stiffness due to better interlayer adhesion and reduced porosity. However, higher layer heights were associated with increased fracture strain, indicating improved ductility but at the expense of reduced strength.

Exposure to a cooling lubricant (CIMTECH A31F) led to progressive degradation of mechanical properties. Prolonged exposure caused a measurable decline in tensile strength, maximum force, and stiffness, likely due to the absorption of moisture and chemical interactions with the PLA matrix. Interestingly, the fracture strain increased with lubricant exposure, suggesting a plasticizing effect that enhanced ductility while compromising strength and rigidity.

### 3.2. Tensile Behavior of PLA+CF

The tensile behavior of FFF 3D-printed PLA+CF specimens was comprehensively evaluated by analyzing the influence of infill density (Section 3.2.1), the number of perimeters (Section 3.2.2), layer height (Section 3.2.3), and exposure to a cooling lubricant (Section 3.2.4).

#### 3.2.1. Effect of Infill Density on the FFF 3D-Printed PLA+CF Specimens

To investigate the effect of infill density on the tensile properties of FFF 3D-printed PLA+CF specimens, three distinct groups of samples were fabricated using a honeycomb internal structure, 0.20 mm layer height, and three perimeters. As presented in Table 8, *Case 11* includes specimens PLA+CF_4302H1 to PLA+CF_4302H5, printed with a 40% infill density. *Case 12* comprises specimens PLA+CF_6302H1 to PLA+CF_6302H5, produced with a 60% infill density, while *Case 13* contains specimens PLA+CF_1302L1 to PLA+CF_1302L5, manufactured with 100% infill density.

The mechanical response of each sample—maximum force, tensile strength, fracture strain, and Young’s modulus—was recorded to assess the influence of increasing infill density on the structural performance of carbon fiber-reinforced PLA under uniaxial tensile loading.

**Figure 11:**
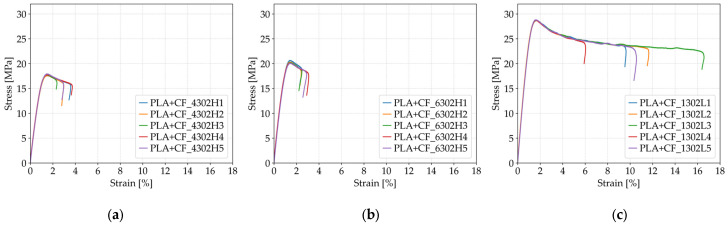
Stress–strain curves of FFF 3D-printed PLA+CF specimens with different infill densities: (**a**) 40% infill (*Case 11*), (**b**) 60% infill (*Case 12*), and (**c**) 100% infill (*Case 13*).

As shown in Figure 11, the tensile strength of PLA+CF specimens increased significantly with higher infill density. At 40% infill (*Case 11*, Figure 11a), the average tensile strength was 17.75 MPa, rising to 20.27 MPa at 60% infill (*Case 12*, Figure 11b), and reaching 28.72 MPa at 100% infill (*Case 13*, Figure 11c). This represents a total improvement of approximately 62% from the lowest to the highest infill configuration. The denser internal structure provided more load-bearing material and improved stress transfer between layers, contributing to a more robust mechanical response under uniaxial tension. The consistency of the tensile strength values is also reflected in the relatively low standard deviations across all cases.

Fracture strain followed a non-linear but overall increasing trend with infill density. The average fracture strain was 3.13% at 40% infill (*Case 11*), slightly decreasing to 2.68% at 60% infill (*Case 12*), and then rising sharply to 10.89% at 100% infill (*Case 13*). The specimens from *Case 13* demonstrated a substantial improvement in ductility, as clearly visible in Figure 11c, where fracture strain values extended well beyond those observed in *Cases 11* and *Cases 12*. This behavior suggests that fully filled PLA+CF structures allow for greater energy absorption and deformation before failure, likely due to improved interlayer bonding and reduced internal voids. While the 60% infill, specimens exhibited slightly lower fracture strain compared to 40%, the sharp increase at full infill confirms that infill density plays a crucial role in modulating both strength and deformation capacity of carbon-fiber-reinforced PLA components.

**Figure 12:**
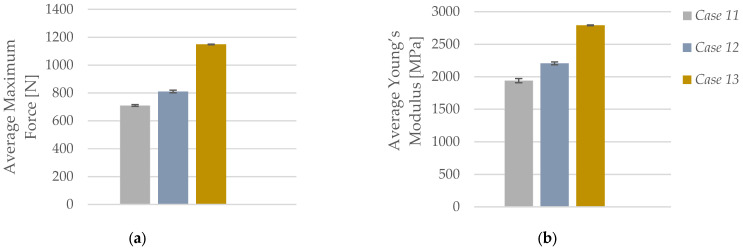
Effect of infill density on the average mechanical properties of FFF 3D-printed PLA+CF specimens: (**a**) average maximum force and (**b**) average Young’s modulus.

As illustrated in Figure 12a, the average maximum force recorded during tensile testing increased substantially with rising infill density in PLA+CF specimens. At 40% infill (*Case 11*), the average maximum force was 710.09 N, which increased to 810.65 N at 60% infill (*Case 12*), and reached 1148.98 N at 100% infill (*Case 13*). This corresponds to a total increase of approximately 62% between the lowest and highest infill configurations. The improved performance is attributed to the greater amount of load-bearing material and the enhanced structural integrity resulting from reduced porosity and better stress transfer within the denser internal structure.

A similar trend is observed for average Young’s modulus, as shown in Figure 12b. The modulus increased from 1939.92 MPa at 40% infill (*Case 11*) to 2206.04 MPa at 60% (*Case 12*), and further to 2791.72 MPa at 100% infill (*Case 13*). This represents a total increase of more than 43% in stiffness. The denser Honeycomb structure at full infill provides superior continuity and reinforcement of the printed layers, which enhances the elastic response under uniaxial tensile loading. These results confirm that increasing infill density not only improves the strength but also significantly enhances the stiffness of carbon fiber-reinforced PLA, making it more suitable for load-bearing applications where both strength and dimensional stability are required.

#### 3.2.2. Effect of Number of Perimeters on the FFF 3D-Printed PLA+CF Specimens

To evaluate the effect of the number of perimeters on the tensile properties of FFF 3D-printed PLA+CF specimens, three groups of samples were fabricated using identical printing parameters: a 60% infill density, 0.20 mm layer height, and *Honeycomb* internal structure. The groups differed only in the number of outer walls (perimeters), as summarized in Table 9.

*Case 14* includes specimens PLA+CF_6102H1 to PLA+CF_6102H5, printed with one perimeter. *Case 15* comprises specimens PLA+CF_6202H1 to PLA+CF_6202H5, produced with two perimeters, while *Case 12* includes specimens PLA+CF_6302H1 to PLA+CF_6302H5, manufactured with three perimeters.

**Figure 13:**
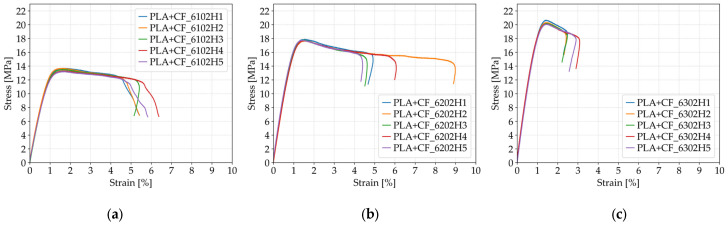
Stress–strain curves of FFF 3D-printed PLA+CF specimens with varying numbers of perimeters: (**a**) one perimeter (*Case 14*), (**b**) two perimeters (*Case 15*), and (**c**) three perimeters (*Case 12*).

As shown in Figure 13, increasing the number of perimeters in PLA+CF specimens significantly affected their tensile behavior. The average tensile strength increased from 13.49 MPa for specimens with one perimeter (*Case 14*, Figure 13a), to 17.76 MPa for those with two perimeters (*Case 15*, Figure 13b), and further to 20.27 MPa for specimens with three perimeters (*Case 12*, Figure 13c). This represents a total improvement of approximately 50% between *Case 14* and *Case 12*. The observed enhancement can be attributed to the increased contribution of outer shell layers, which improve the load distribution and delay the onset of fracture initiation under uniaxial tensile loading.

The trend in fracture strain is more complex. Specimens with one perimeter (*Case 14*) exhibited an average fracture strain of 5.69%, which slightly increased to 5.80% in *Case 15* (two perimeters), and then decreased to 2.68% in *Case 12* (three perimeters). As observed in Figure 13, particularly in subfigures (a) and (b), the specimens with fewer perimeters allowed for greater elongation before failure, indicating enhanced ductility. In contrast, the reduced fracture strain in *Case 12* suggests that the additional shell layers, while increasing strength and stiffness, may reduce flexibility and lead to earlier failure once peak stress is reached. These findings point to a mechanical trade-off, where increasing the number of perimeters improves tensile strength at the cost of reduced ductility in carbon fiber-reinforced PLA specimens.

**Figure 14:**
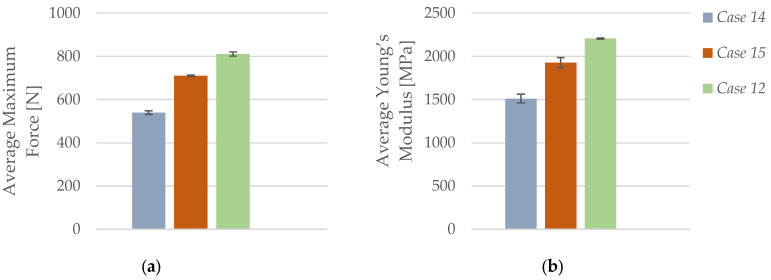
Effect of the number of perimeters on the average mechanical properties of FFF 3D-printed PLA+CF specimens: (**a**) average maximum force and (**b**) average Young’s modulus.

As illustrated in Figure 14a, the average maximum force increased progressively with a higher number of perimeters. Specimens with one perimeter (*Case 14*) reached an average maximum force of 539.51 N, which rose to 710.53 N for two perimeters (*Case 15*), and further to 810.65 N for three perimeters (*Case 12*). This represents an overall improvement of approximately 50%, confirming that additional outer walls enhance the load-bearing capacity of carbon fiber-reinforced PLA structures by increasing surface reinforcement and improving structural stability under tensile stress.

A similar trend is evident in Figure 14b, which shows the corresponding average Young’s modulus values. The stiffness increased from 1511.76 MPa in *Case 14* to 1928.28 MPa in *Case 15* and peaked at 2206.04 MPa in *Case 12*. The overall increase of more than 45% in elastic modulus reflects the role of additional perimeters in enhancing rigidity by reducing localized deformation. The thicker outer shell improves interlayer bonding and restricts strain localization, resulting in greater resistance to elastic elongation. These findings indicate that increasing the number of perimeters is an effective strategy for improving both strength and stiffness in PLA+CF parts, particularly for load-bearing applications where dimensional stability is critical.

#### 3.2.3. Effect of Layer Height on the FFF 3D-Printed PLA+CF Specimens

To investigate the effect of layer height on the tensile properties of FFF 3D-printed PLA+CF specimens, three sets of samples were fabricated using identical process parameters—60% infill density, *Honeycomb* internal structure, and two perimeters—while varying only the layer height. As summarized in Table 10, *Case 15* includes specimens PLA+CF_6202H1 to PLA+CF_6202H5, printed with a layer height of 0.20 mm. *Case 16* comprises specimens PLA+CF_6203H1 to PLA+CF_6203H5, printed with a 0.30 mm layer height, while *Case 17* includes specimens PLA+CF_6304H1 to PLA+CF_6304H5, fabricated with a 0.40 mm layer height.

The results in Table 10 reveal that tensile strength of the PLA+CF specimens exhibits a decreasing trend with increasing layer height. At 0.20 mm layer height (*Case 15*), the average tensile strength reached 17.76 MPa. This value declined to 16.52 MPa at 0.30 mm (*Case 16*) and remained relatively stable at 17.23 MPa for the 0.40 mm layer height (*Case 17*). Although the change between *Case 16* and *Case 17* is minor, the strength reduction from the finest to the coarsest layer resolution (~7%) suggests that smaller layer heights facilitate better interlayer bonding and contribute to improved structural integrity under tensile stress. The slightly higher average tensile strength in *Case 17* compared to *Case 16* may be influenced by local microstructural variations or stochastic effects in fiber orientation.

**Figure 15:**
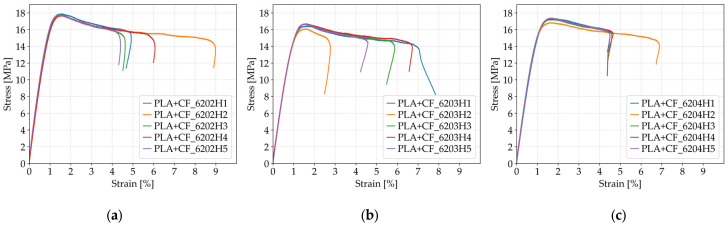
Stress–strain curves of FFF 3D-printed PLA+CF specimens with different layer heights: (**a**) 0.20 mm (*Case 15*), (**b**) 0.30 mm (*Case 16*), and (**c**) 0.40 mm (*Case 17*).

The graphical representations of the strength and fracture strain for individual FFF 3D-printed PLA+CF tensile-tested specimens are shown in Figure 15.

In terms of fracture strain, the relationship with layer height is less linear. The average fracture strain was 5.80% at 0.20 mm (*Case 15*), decreased slightly to 5.56% at 0.30 mm (*Case 16*), and increased again to 5.03% at 0.40 mm (*Case 17*). Although the differences are relatively small and within the range of statistical variation, it is notable that all three cases demonstrate moderate ductility. The overall data suggest that while higher layer heights might result in slightly lower tensile strength, they do not drastically compromise the ductility of PLA+CF specimens. The relatively stable fracture strain values across the tested range indicate that the carbon fiber reinforcement helps maintain dimensional stability and fracture strain tolerance even when layer resolution is reduced.

**Figure 16:**
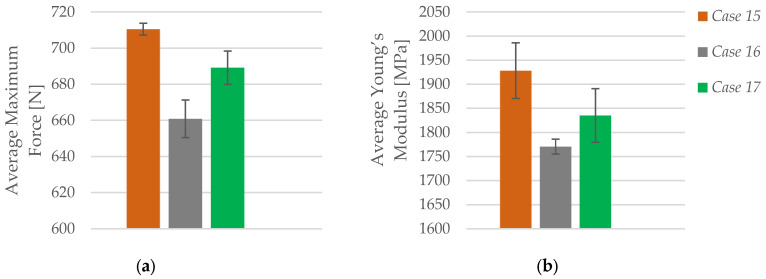
Effect of layer height on the average mechanical properties of FFF 3D-printed PLA+CF specimens: (**a**) average maximum force, and (**b**) average Young’s modulus.

As illustrated in Figure 16a, the average maximum force recorded during tensile testing shows a decreasing trend with increasing layer height. Specimens printed with a 0.20 mm layer height (*Case 15*) achieved the highest average force of 710.53 N. This value dropped to 660.85 N for specimens printed at 0.30 mm (*Case 16*), and slightly increased again to 689.12 N at 0.40 mm (*Case 17*). Despite the partial recovery in *Case 17*, the overall trend confirms that finer layer resolution generally leads to improved load-bearing capacity, likely due to enhanced interlayer bonding and lower void content.

Similarly, the results for the average Young’s modulus (Figure 16b) follow the same trend. The highest average modulus was observed in *Case 15* (1928.28 MPa), followed by 1835.05 MPa in *Case 17*, and the lowest in *Case 16* (1770.47 MPa). The reduced stiffness at coarser layer heights reflects diminished interlayer cohesion and increased anisotropy in the printed structure. Although the modulus in *Case 17* slightly exceeds that of *Case 16*, the overall pattern suggests that lower layer heights are more effective for achieving higher stiffness in carbon fiber-reinforced PLA, reinforcing the importance of fine layer resolution in precision-demanding applications.

#### 3.2.4. Effect of Lubricant Exposure on the FFF 3D-Printed PLA+CF Specimens

To investigate the effect of lubricant exposure on the tensile properties of FFF 3D-printed PLA+CF specimens, three sets of samples were fabricated using identical process parameters—60% infill density, *Honeycomb* internal structure, 0.20 mm layer height, and three perimeters—while varying only the duration of exposure to a water-based cooling lubricant (CIMTECH A31F). The resulting tensile properties—maximum force, tensile strength, fracture strain, and Young’s modulus—are summarized in Table 11.

*Case 12* includes specimens PLA+CF_6302H1 to PLA+CF_6302H5, which were not exposed to lubricant and serve as the reference group. *Case 18* comprises specimens PLA+CF_6302H71 to PLA+CF_6302H75, which were exposed to the lubricant for 7 days, while *Case 19* includes specimens PLA+CF_6302H31 to PLA+CF_6302H35, which were exposed for 30 days. Specimen codes reflect the exposure status and ensure full traceability. The mechanical performance of the samples was evaluated to assess the degradation effects and material response under different durations of environmental exposure.

**Figure 17:**
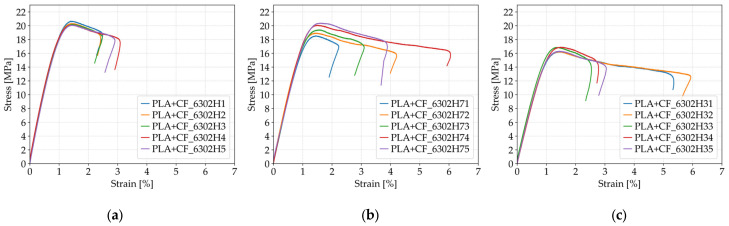
Stress–strain curves of FFF 3D-printed PLA+CF specimens subjected to different durations of lubricant exposure: (**a**) unexposed (*Case 12*), (**b**) exposed for 7 days (*Case 18*), and (**c**) exposed for 30 days (*Case 19*).

The stress–strain behavior of PLA+CF specimens subjected to different durations of lubricant exposure is illustrated in Figure 17. The control group (*Case 12*), which was not exposed to lubricants, shows the highest tensile strength and stiffness with limited fracture strain. After 7 days of exposure (*Case 18*), a moderate decrease in tensile strength is observed, accompanied by a noticeable increase in ductility. Prolonged exposure for 30 days (*Case 19*) results in further strength reduction and slightly lower stiffness, while fracture strain remains elevated. These trends confirm the progressive impact of environmental degradation on the mechanical performance of PLA+CF materials in lubricated conditions.

As presented in Table 11 and illustrated in Figure 17, the tensile strength of PLA+CF specimens decreased with longer exposure to the CIMTECH A31F lubricant. The unexposed group (*Case 12*) exhibited the highest average tensile strength at 20.27 MPa. After 7 days of exposure (*Case 18*), the average strength declined to 19.47 MPa and dropped further to 16.51 MPa after 30 days of exposure (*Case 19*). This represents a total reduction of approximately 19%, indicating a gradual degradation of the material’s load-bearing capability due to environmental influence. The weakening is likely caused by the lubricant’s penetration and partial chemical interaction with the PLA matrix, which compromises interlayer bonding and fiber–matrix adhesion.

The trend in fracture strain shows a distinct increase following short-term exposure and a partial decline with prolonged exposure. The unexposed specimens (*Case 12*) had the lowest average fracture strain at 2.68%, which rose significantly to 3.90% after 7 days (*Case 18*). After 30 days (*Case 19*), the fracture strain remained elevated at 3.93%, although with increased variability. As visualized in Figure 17, the lubricant exposure induced a softening or plasticizing effect, enabling greater deformation before failure. However, the simultaneous reduction in tensile strength suggests that this gain in ductility is accompanied by structural weakening. These findings confirm that PLA+CF composites are sensitive to moisture-based lubricants, and exposure duration is a key factor in balancing strength and flexibility in practical applications.

**Figure 18:**
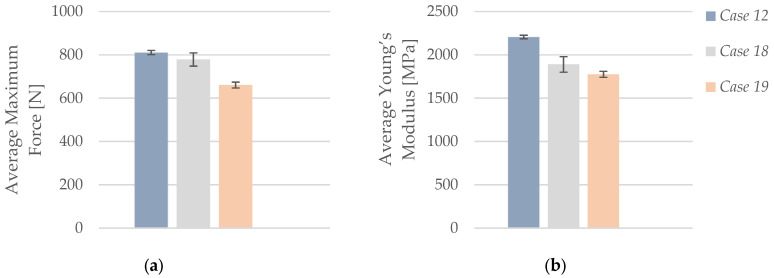
Effect of lubricant exposure duration on the average mechanical properties of FFF 3D-printed PLA+CF specimens: (**a**) average maximum force, and (**b**) average Young’s modulus.

As shown in Figure 18a, the average maximum force exhibited a clear declining trend with increasing duration of lubricant exposure. The unexposed specimens (*Case 12*) achieved the highest average force of 810.65 N, which dropped to 778.64 N after 7 days of exposure (*Case 18*), and further declined to 660.41 N following 30 days of immersion (*Case 19*). This represents an overall reduction of nearly 19% in load-bearing capacity between the reference and the most degraded group. The progressive decline suggests that extended contact with the CIMTECH A31F lubricant weakens the material structure, likely due to water absorption and matrix–fiber interface deterioration.

A similar trend is observed in Figure 18b for the average Young’s modulus, which quantifies the stiffness of the material. The modulus decreased from 2206.04 MPa in *Case 12* to 1890.11 MPa in *Case 18*, and further to 1775.10 MPa in *Case 19*. The total reduction of approximately 20% highlights a significant loss in elastic resistance over time. This decrease can be attributed to plasticizing effects caused by moisture ingress and chemical interactions that disrupt interlayer cohesion. These findings confirm that even short-term lubricant exposure can measurably affect both the strength and stiffness of PLA+CF components, underlining the importance of environmental durability in demanding engineering applications.

#### 3.2.5. Summary of PLA+CF Mechanical Response

The mechanical response of FFF 3D-printed PLA+CF was comprehensively evaluated under four distinct experimental factors: infill density, number of perimeters, layer height, and exposure to a water-based cooling lubricant (CIMTECH A31F). The results consistently demonstrate that the examined parameters significantly influence the tensile behavior of PLA+CF, particularly in terms of strength, stiffness, and ductility.

An increase in infill density from 40% to 100% (*Cases 11–13*) led to marked improvements in both tensile strength (from 17.75 MPa to 28.72 MPa) and Young’s modulus (from 1939.92 MPa to 2791.72 MPa). This performance enhancement is attributed to reduced internal porosity and improved fiber–matrix continuity, which collectively promote more effective stress distribution. A notable increase in fracture strain was also observed at full infill, indicating not only greater strength but also improved energy absorption.

Similarly, increasing the number of perimeters from one to three (*Cases 12–14*) resulted in significant gains in maximum force and stiffness, with tensile strength rising from 13.49 MPa to 20.27 MPa and Young’s modulus increasing from 1511.76 MPa to 2206.04 MPa. The additional shell layers enhanced load-bearing capacity and restricted localized deformation, although excessive shell thickness slightly reduced ductility.

In contrast, the effect of layer height (*Cases 15–17*) revealed a trade-off between precision and mechanical performance. Specimens printed with finer resolution (0.20 mm) achieved the highest tensile strength (17.76 MPa) and stiffness (1928.28 MPa), while higher layer heights (0.30–0.40 mm) resulted in a moderate decline in both parameters. Despite this, fracture strain values remained relatively stable, suggesting that ductility is less sensitive to vertical resolution in PLA+CF than in unreinforced PLA.

Lubricant exposure (*Cases 12*, *18*, *and 19*) had a degrading effect on all mechanical properties. Prolonged immersion in the lubricant caused an approximate 19% drop in tensile strength and a 20% reduction in Young’s modulus. While the fracture strain increased due to a plasticizing effect, the accompanying loss in structural integrity and stiffness highlights the vulnerability of PLA+CF to moisture-based environments.

In summary, PLA+CF exhibits superior mechanical performance compared to pure PLA, particularly at high infill densities and with additional shell layers. However, its performance is sensitive to environmental conditions, especially prolonged exposure to lubricants. The results underline the importance of optimizing both structural and environmental parameters when designing PLA+CF components for engineering applications.

## 4. Discussion

The comparative mechanical analysis of PLA and PLA+CF specimens across varying infill density, number of perimeters, layer height, and lubricant exposure reveals distinct material-dependent trends in tensile strength and elastic modulus. While both materials responded similarly to the tested parameters in terms of directionality (e.g., strength increasing with infill), the magnitude of their responses and the underlying mechanisms differed due to the presence of carbon fiber reinforcement in PLA+CF.

**Figure 19:**
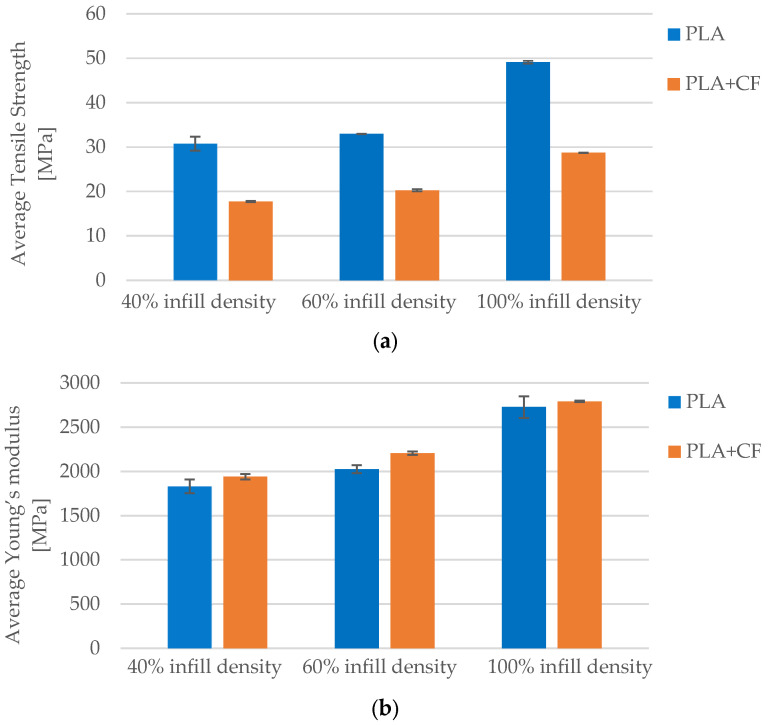
Effect of infill density on the average mechanical properties of FFF 3D-printed PLA and PLA+CF specimens: (**a**) average tensile strength, and (**b**) average Young’s modulus.

Infill density had the most pronounced effect on tensile properties. As infill increased from 40% to 100%, the tensile strength of PLA improved from 30.75 MPa to 49.11 MPa, representing a 60% increase. In parallel, PLA+CF samples exhibited an increase from 17.75 MPa to 28.72 MPa—an enhancement of approximately 62%. Young’s modulus followed a similar trend: PLA rose from 1832.71 MPa to 2727.08 MPa (+49%), while PLA+CF increased from 1939.92 MPa to 2791.72 MPa (+44%). These results, shown in Figure 19, indicate that although PLA exhibited higher strength values overall, PLA+CF consistently provided greater stiffness, underlining the reinforcing effect of carbon fibers. Moreover, the superior modulus gain in PLA+CF suggests that the mechanical efficiency of the honeycomb structure is further enhanced by the presence of fibers when internal volume is maximized.

**Figure 20:**
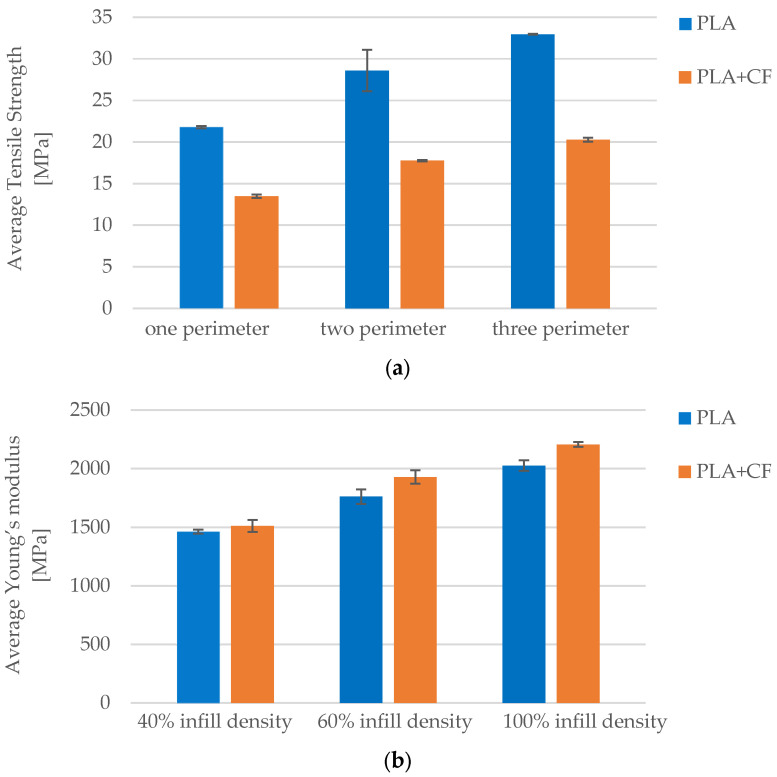
Effect of number of perimeters on the average mechanical properties of FFF 3D-printed PLA and PLA+CF specimens: (**a**) average tensile strength, and (**b**) average Young’s modulus.

The number of perimeters also contributed significantly to mechanical performance. Increasing the perimeter count from 1 to 3 resulted in tensile strength gains of 51% in PLA (from 21.79 MPa to 32.94 MPa) and 50% in PLA+CF (from 13.49 MPa to 20.27 MPa). Young’s modulus increased from 1461.99 MPa to 2025.72 MPa in PLA (+38%) and from 1511.76 MPa to 2206.04 MPa in PLA+CF (+46%). As shown in Figure 20, these improvements are attributed to improved structural integrity provided by the outer shell, which helps redistribute stress away from infill regions. PLA+CF benefited more in terms of stiffness, likely due to fiber alignment near the surface, which enhances rigidity under axial loading.

**Figure 21:**
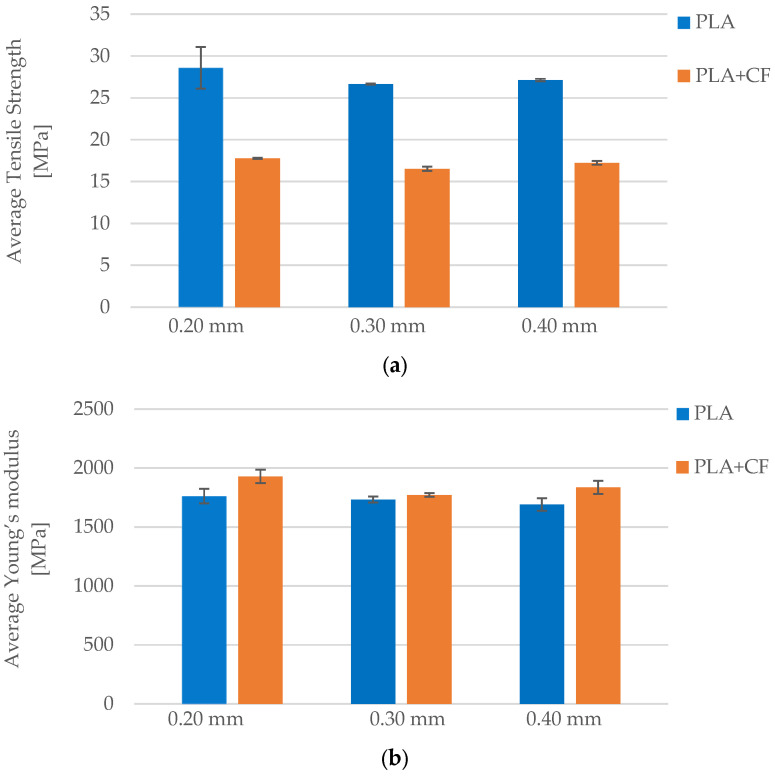
Effect of layer height on the average mechanical properties of FFF 3D-printed PLA and PLA+CF specimens: (**a**) average tensile strength, and (**b**) average Young’s modulus.

The effect of layer height on the mechanical performance of FFF 3D-printed specimens was moderate but clearly quantifiable. In PLA, increasing the layer height from 0.20 mm to 0.40 mm resulted in a reduction in tensile strength by approximately 22%, from 28.59 MPa to 22.28 MPa, and a decline in Young’s modulus by about 4%, from 1761.87 MPa to 1690.10 MPa. In contrast, PLA+CF exhibited a less pronounced decrease in tensile strength, dropping by only ~3%, from 17.76 MPa to 17.23 MPa, while its modulus declined by ~5%, from 1928.28 MPa to 1835.05 MPa. These differences, illustrated in Figure 21a,b, suggest that PLA is more sensitive to interlayer resolution, with greater dependency on cohesive bonding between thinner layers. In comparison, PLA+CF’s performance remained more stable, likely due to the bridging effect of carbon fibers across adjacent layers, which mitigates the loss of mechanical continuity caused by thicker deposition heights. These findings are in line with previous studies such as Ref. [22], which highlighted the impact of vertical resolution on bonding quality and mechanical uniformity in PLA.

**Figure 22:**
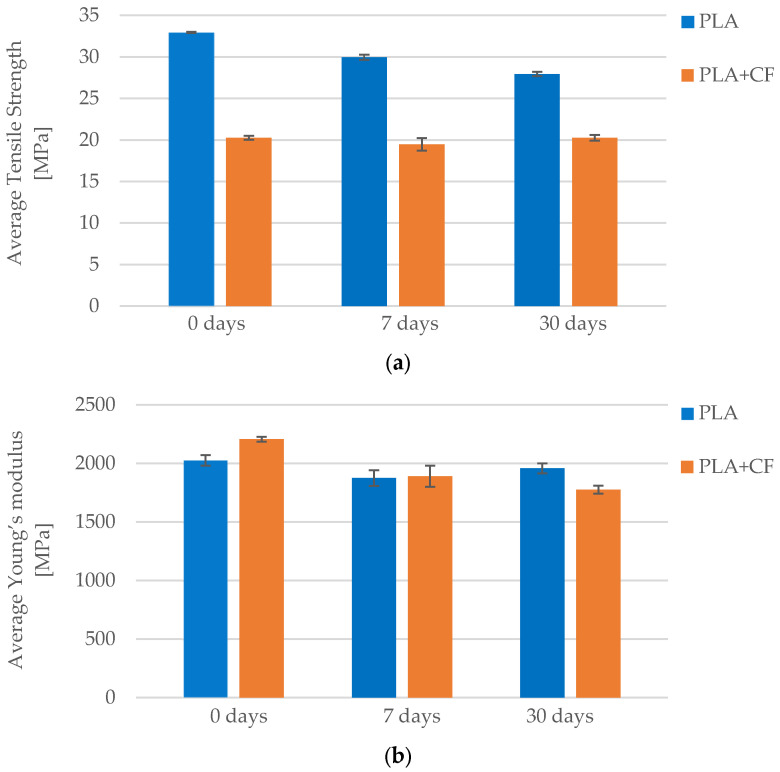
Effect of lubricant exposure on the average mechanical properties of FFF 3D-printed PLA and PLA+CF specimens: (**a**) average tensile strength, and (**b**) average Young’s modulus.

Prolonged exposure to a cooling lubricant (CIMTECH A31F) resulted in observable degradation of mechanical properties in both materials. For PLA, the tensile strength decreased by approximately 15%, from 32.94 MPa (unexposed) to 27.94 MPa after 30 days of exposure. PLA+CF exhibited a greater loss of about 19%, decreasing from 20.27 MPa to 16.51 MPa. Similarly, Young’s modulus declined from 2025.72 MPa to 1957.08 MPa in PLA (~3% decrease), while PLA+CF dropped from 2206.04 MPa to 1775.10 MPa, representing a more significant ~20% reduction. These trends, as shown in Figure 22a,b, indicate that PLA+CF is more vulnerable to stiffness degradation under environmental exposure, likely due to microstructural disruption at the fiber–matrix interface.

Such degradation trends are in line with previous findings reported in Refs. [18,34], where similar moisture-induced weakening of fiber-reinforced structures was observed under lubricated or humid conditions. Interestingly, PLA displayed a notable increase in fracture strain, suggesting plasticization effects and increased ductility following moisture absorption, while PLA+CF remained relatively dimensionally stable but more brittle. This contrast underscores the material-specific degradation behavior, relevant for applications subjected to lubricated or humid environments.

## 5. Conclusions

This study systematically evaluated the tensile behavior of FFF 3D-printed PLA and PLA+CF materials under the influence of four key parameters: infill density, number of perimeters, layer height, and exposure to a water-based cooling lubricant (CIMTECH A31F). The findings confirm that both geometric and environmental parameters significantly affect the mechanical performance of printed parts, and that the response is material-dependent due to differences in polymer composition and reinforcement phase.

The results clearly show that infill density has the most dominant influence on tensile strength and stiffness, with both materials exhibiting over 60% improvement in tensile strength from 40% to 100% infill. These trends align well with findings by Lanzotti et al. [24] and Magri et al. [19], who also reported infill as the most critical parameter for strength optimization in FFF-printed PLA and CF-reinforced PLA. PLA+CF showed higher stiffness in all infill cases, corroborating the reinforcement effect described in studies by Papageorgiou et al. [20] and Zhi et al. [30].

Increasing the number of perimeters significantly enhanced tensile properties, particularly stiffness, with PLA+CF showing a 46% increase in Young’s modulus from 1 to 3 walls. This agrees with conclusions from Torres et al. [25] and Syrlybayev et al. [21], who emphasized the perimeter shell’s contribution to stress redistribution and crack propagation resistance in multi-material systems.

Reducing the layer height led to increased strength and stiffness in both materials, with PLA being more sensitive to vertical resolution changes than PLA+CF. These findings support those of Kiendl and Gao [22], who demonstrated that finer layers improve interlayer adhesion and reduce anisotropy. However, the lower sensitivity of PLA+CF suggests that carbon fiber networks help maintain mechanical integrity across layer interfaces, echoing observations from Tian et al. [28] and Camargo et al. [36].

Finally, prolonged lubricant exposure caused degradation in both strength and stiffness, with PLA+CF losing 19.5% of its stiffness after 30 days. While similar moisture-induced degradation was reported in the work of Kim et al. [34] and Fang et al. [33], our findings further demonstrate that fiber reinforcement can mitigate some loss in dimensional stability, albeit at the expense of ductility. Notably, PLA showed a greater increase in fracture strain after exposure, consistent with the plasticizing behavior described by Naveed [17] and Dizon et al. [26].

While this study provides valuable insights into the tensile behavior of FFF 3D-printed PLA and PLA+CF under varying process parameters and environmental exposure, several limitations should be acknowledged. Firstly, the experimental matrix was restricted to selected infill densities, perimeter numbers, and layer heights, which may not fully capture the complexity of all possible parameter interactions. Additionally, only tensile properties were evaluated; other mechanical characteristics such as flexural strength, impact resistance, and long-term durability were not addressed. The lubricant exposure tests were limited to a single coolant type and duration, which may not represent all industrial scenarios.

Researchers should therefore exercise caution when generalizing these findings to different materials, parameter ranges, or environmental conditions. Future studies are encouraged to expand the parameter space, incorporate additional mechanical tests, and evaluate the influence of various lubricant types and exposure times. Despite these limitations, the current work offers a robust foundation for further research and process optimization in the additive manufacturing of polymer composites.

In summary, PLA offers higher tensile strength and greater ductility, making it suitable for applications requiring energy absorption and flexibility. PLA+CF, on the other hand, exhibits superior stiffness and environmental resistance, which are advantageous for structural components subjected to static loading and potential fluid exposure. These insights provide valuable guidelines for parameter selection in functional design and manufacturing of polymer-based and composite 3D-printed parts. Future research should investigate cyclic loading, fatigue resistance, and multi-environmental aging to further assess the long-term applicability of these materials in service conditions.

## Figures and Tables

**Table 1:** Manufacturer-provided specifications of PLA and PLA+CF materials, adapted from Ref. [58].

Material Type	PLA	PLA+CF
Filament diameter [mm]	1.75	1.75
Net filament weight [g]	1000	1000
Printing speed [mm/s]	40–250	40–250
Drying conditions before printing	55 °C, 8 h	60 °C, 5 h
Layer thickness [mm]	0.12–0.4	0.12–0.4
Nozzle temperature [°C]	190–240	200–230
Build plate temperature [°C]	25–60	25–60
Cooling fan [%]	50–100	50–100
Retraction length [mm]	1–2	1–2
Retraction speed [mm/s]	30–50	30–50
Printing and storage humidity	40 °C, ≤20% RH	40 °C, ≤20% RH

**Table 2:** Manufacturer-provided mechanical parameters of PLA and PLA+CF materials, adapted from Refs. [58,59,60,61].

Parameters	Test Method	PLA	PLA+CF
Density [g/cm^3^]	ISO 1183 [59]	1.25–1.26	1.26–1.27
Young’s modulus [MPa]	ISO 527 [60]	1000–1100	1100–1300
Tensile strength [MPa]	ISO 527 [60]	45–49	40–45
Elongation at break [%]	ISO 527 [60]	13.5–15.5	11.5–13.5
Water absorption, 23 °C, 24 h [%]	ISO 62 [62]	<0.3	0.12–0.3
Heat deflection temperature [°C]	ISO 75 [61]	53	60

**Table 4:** Tensile test results for PLA specimens—Effect of Infill Density.

Case	Specimen Code	Max. Force [N]	Tensile Strength [MPa]	Fracture Strain [%]	Young’s Modulus [MPa]
*Case 1*	PLA_4302H1	1199.48	29.98	3.64	1701.84
	PLA_4302H2	1200.94	30.02	4.26	1858.06
	PLA_4302H3	1203.31	30.08	3.12	1890.89
	PLA_4302H4	1342.40	33.56	3.69	1825.94
	PLA_4302H5	1204.77	30.12	4.81	1886.79
	*Average*	*1230.18*	*30.75*	*3.92*	*1832.71*
	*St. Dev.*	*62.77*	*1.57*	*0.62*	*77.66*
*Case 2*	PLA_6302H1	1316.25	32.90	4.78	2037.56
	PLA_6302H2	1319.37	32.98	5.54	2000.98
	PLA_6302H3	1321.47	33.03	3.63	2098.91
	PLA_6302H4	1316.14	32.90	4.93	1992.79
	PLA_6302H5	1315.30	32.88	4.66	1998.78
	*Average*	*1317.71*	*32.94*	*4.71*	*2025.72*
	*St. Dev.*	*2.61*	*0.06*	*0.69*	*44.55*
*Case 3*	PLA_1302L1	1962.94	49.07	6.77	2732.53
	PLA_1302L2	1971.77	49.29	5.86	2521.40
	PLA_1302L3	1964.88	49.12	5.73	2848.32
	PLA_1302L4	1945.04	48.62	7.29	2762.45
	PLA_1302L5	1978.46	49.46	7.27	2770.69
	*Average*	*1964.62*	*49.11*	*6.58*	*2727.08*
	*St. Dev.*	*12.54*	*0.31*	*0.75*	*122.66*

**Table 5:** Tensile test results for PLA specimens—Effect of Number of Perimeters.

Case	Specimen Code	Max. Force [N]	Tensile Strength [MPa]	Fracture Strain [%]	Young’s Modulus [MPa]
*Case 4*	PLA_6102H1	863.57	21.59	3.39	1492.99
	PLA_6102H2	869.07	21.73	4.08	1452.13
	PLA_6102H3	878.49	21.96	4.36	1451.19
	PLA_6102H4	874.87	21.87	4.40	1455.76
	PLA_6102H5	872.99	21.82	4.42	1457.87
	*Average*	*871.79*	*21.79*	*4.13*	*1461.99*
	*St. Dev.*	*5.72*	*0.14*	*0.43*	*17.54*
*Case 5*	PLA_6202H1	1091.31	27.28	3.07	1571.23
	PLA_6202H2	1095.91	27.39	2.85	1765.80
	PLA_6202H3	1321.79	33.03	3.52	1696.18
	PLA_6202H4	1097.79	27.44	3.01	1733.32
	PLA_6202H5	1112.93	27.82	3.20	1862.81
	*Average*	*1143.89*	*28.59*	*3.13*	*1761.87*
	*St. Dev.*	*99.62*	*2.49*	*0.25*	*62.14*
*Case 6*	PLA_6302H1	1316.25	32.90	4.78	2037.56
	PLA_6302H2	1319.37	32.98	5.54	2000.98
	PLA_6302H3	1321.47	33.03	3.63	2098.91
	PLA_6302H4	1316.14	32.90	4.93	1992.79
	PLA_6302H5	1315.30	32.88	4.66	1998.78
	*Average*	*1317.71*	*32.94*	*4.71*	*2025.72*
	*St. Dev.*	*2.61*	*0.06*	*0.69*	*44.55*

**Table 6:** Tensile test results for PLA specimens—effect of layer height.

Case	Specimen Code	Max. Force [N]	Tensile Strength [MPa]	Fracture Strain [%]	Young’s Modulus [MPa]
*Case 5*	PLA_6202H1	1091.31	27.28	3.07	1571.23
	PLA_6202H2	1095.91	27.39	2.85	1765.80
	PLA_6202H3	1321.79	33.03	3.52	1696.18
	PLA_6202H4	1097.79	27.44	3.01	1733.32
	PLA_6202H5	1112.93	27.82	3.20	1862.81
	*Average*	*1143.89*	*28.59*	*3.13*	*1761.87*
	*St. Dev.*	*99.62*	*2.49*	*0.25*	*62.14*
*Case 7*	PLA_6203H1	1062.01	26.55	3.76	1748.98
	PLA_6203H2	1067.11	26.68	4.12	1749.28
	PLA_6203H3	1070.18	26.75	3.67	1747.73
	PLA_6203H4	1066.08	26.65	3.78	1722.49
	PLA_6203H5	1064.02	26.60	3.92	1691.57
	*Average*	*1065.88*	*26.65*	*3.85*	*1732.01*
	*St. Dev.*	*3.09*	*0.08*	*0.18*	*25.29*
*Case 8*	PLA_6204H1	1077.05	26.93	4.83	1712.37
	PLA_6204H2	1089.32	27.23	5.19	1679.23
	PLA_6204H3	1089.98	27.25	4.07	1762.74
	PLA_6204H4	1087.73	27.19	4.89	1676.65
	PLA_6204H5	1081.28	27.03	5.19	1619.52
	*Average*	*1085.07*	*27.13*	*4.83*	*1690.10*
	*St. Dev.*	*5.66*	*0.14*	*0.46*	*52.56*

**Table 7:** Tensile test results for PLA specimens—effect of lubricant exposure.

Case	Specimen Code	Max. Force [N]	Tensile Strength [MPa]	Fracture Strain [%]	Young’s Modulus [MPa]
*Case 6*	PLA_6302H1	1316.25	32.90	4.78	2037.56
	PLA_6302H2	1319.37	32.98	5.54	2000.98
	PLA_6302H3	1321.47	33.03	3.63	2098.91
	PLA_6302H4	1316.14	32.90	4.93	1992.79
	PLA_6302H5	1315.30	32.88	4.66	1998.78
	*Average*	*1317.71*	*32.94*	*4.71*	*2025.72*
	*St. Dev.*	*2.61*	*0.06*	*0.69*	*44.55*
*Case 9*	PLA_6302H71	1184.89	29.62	7.37	1882.49
	PLA_6302H72	1208.05	30.20	7.19	1798.84
	PLA_6302H73	1187.75	29.69	7.05	1867.71
	PLA_6302H74	1197.51	29.94	7.79	1931.67
	PLA_6302H75	1214.66	30.37	8.71	1952.85
	*Average*	*1198.57*	*29.96*	*7.63*	*1874.71*
	*St. Dev.*	*12.78*	*0.32*	*0.67*	*66.86*
*Case 10*	PLA_6302H31	1127.25	28.18	6.69	2005.43
	PLA_6302H32	1105.33	27.63	7.38	1942.88
	PLA_6302H33	1116.08	27.90	7.49	1924.38
	PLA_6302H34	1130.20	28.25	7.71	1914.70
	PLA_6302H35	1110.02	27.75	7.32	1997.99
	*Average*	*1117.78*	*27.94*	*7.32*	*1957.08*
	*St. Dev.*	*10.75*	*0.27*	*0.38*	*42.07*

**Table 8:** Tensile test results for PLA+CF specimens—effect of infill density.

Case	Specimen Code	Max. Force [N]	Tensile Strength [MPa]	Fracture Strain [%]	Young’s Modulus [MPa]
*Case 11*	PLA+CF_4302H1	704.17	17.60	3.59	1902.54
	PLA+CF_4302H2	704.67	17.62	2.98	1935.49
	PLA+CF_4302H3	711.62	17.79	2.37	1980.81
	PLA+CF_4302H4	711.43	17.78	3.74	1919.77
	PLA+CF_4302H5	718.56	17.96	2.96	1961.00
	*Average*	*710.09*	*17.75*	*3.13*	*1939.92*
	*St. Dev.*	*5.92*	*0.15*	*0.55*	*31.38*
*Case 12*	PLA+CF_6302H1	826.27	20.66	2.49	2240.74
	PLA+CF_6302H2	813.91	20.32	2.42	2199.22
	PLA+CF_6302H3	807.90	20.20	2.48	2205.80
	PLA+CF_6302H4	803.24	20.08	3.09	2198.08
	PLA+CF_6302H5	802.91	20.07	2.91	2186.36
	*Average*	*810.65*	*20.27*	*2.68*	*2206.04*
	*St. Dev.*	*9.64*	*0.24*	*0.30*	*20.62*
*Case 13*	PLA+CF_1302L1	1146.84	28.67	9.64	2792.27
	PLA+CF_1302L2	1147.27	28.68	11.66	2785.10
	PLA+CF_1302L3	1149.48	28.74	16.58	2790.18
	PLA+CF_1302L4	1148.92	28.72	6.04	2803.75
	PLA+CF_1302L5	1152.39	28.81	10.56	2787.27
	*Average*	*1148.98*	*28.72*	*10.89*	*2791.72*
	*St. Dev.*	*2.20*	*0.05*	*3.81*	*7.26*

**Table 9:** Tensile test results for PLA+CF specimens—effect of number of perimeters.

Case	Specimen Code	Max. Force [N]	Tensile Strength [MPa]	Fracture Strain [%]	Young’s Modulus [MPa]
*Case 14*	PLA+CF_6102H1	547.62	13.69	5.42	1554.47
	PLA+CF_6102H2	547.14	13.68	5.41	1569.47
	PLA+CF_6102H3	540.65	13.52	5.41	1514.20
	PLA+CF_6102H4	532.27	13.31	6.38	1459.82
	PLA+CF_6102H5	529.84	13.25	5.83	1460.85
	*Average*	*539.51*	*13.49*	*5.69*	*1511.76*
	*St. Dev.*	*8.23*	*0.21*	*0.43*	*51.11*
*Case 15*	PLA+CF_6202H1	715.66	17.89	4.92	1992.85
	PLA+CF_6202H2	707.12	17.68	8.98	1900.07
	PLA+CF_6202H3	711.04	17.77	4.63	1882.19
	PLA+CF_6202H4	708.37	17.71	6.07	1877.88
	PLA+CF_6202H5	710.45	17.76	4.40	1988.40
	*Average*	*710.53*	*17.76*	*5.80*	*1928.28*
	*St. Dev.*	*3.27*	*0.08*	*1.89*	*57.54*
*Case 12*	PLA+CF_6302H1	826.27	20.66	2.49	2240.74
	PLA+CF_6302H2	813.91	20.32	2.42	2199.22
	PLA+CF_6302H3	807.90	20.20	2.48	2205.80
	PLA+CF_6302H4	803.24	20.08	3.09	2198.08
	PLA+CF_6302H5	802.91	20.07	2.91	2186.36
	*Average*	*810.65*	*20.27*	*2.68*	*2206.04*
	*St. Dev.*	*9.64*	*0.24*	*0.30*	*20.62*

**Table 10:** Tensile test results for PLA+CF specimens—effect of layer height.

Case	Specimen Code	Max. Force [N]	Tensile Strength [MPa]	Fracture Strain [%]	Young’s Modulus [MPa]
*Case 15*	PLA+CF_6202H1	715.66	17.89	4.92	1992.85
	PLA+CF_6202H2	707.12	17.68	8.98	1900.07
	PLA+CF_6202H3	711.04	17.77	4.63	1882.19
	PLA+CF_6202H4	708.37	17.71	6.07	1877.88
	PLA+CF_6202H5	710.45	17.76	4.40	1988.40
	*Average*	*710.53*	*17.76*	*5.80*	*1928.28*
	*St. Dev.*	*3.27*	*0.08*	*1.89*	*57.54*
*Case 16*	PLA+CF_6203H1	657.64	16.44	7.85	1787.41
	PLA+CF_6203H2	643.89	16.10	2.76	1767.12
	PLA+CF_6203H3	667.89	16.70	5.88	1780.37
	PLA+CF_6203H4	667.86	16.70	6.72	1747.08
	PLA+CF_6203H5	666.96	16.67	4.58	1770.35
	*Average*	*660.85*	*16.52*	*5.56*	*1770.47*
	*St. Dev.*	*10.41*	*0.26*	*1.96*	*15.35*
*Case 17*	PLA+CF_6304H1	693.39	17.34	4.50	1783.13
	PLA+CF_6304H2	673.25	16.83	6.89	1895.25
	PLA+CF_6304H3	689.02	17.23	4.59	1872.83
	PLA+CF_6304H4	694.11	17.35	4.65	1769.50
	PLA+CF_6304H5	695.84	17.40	4.50	1854.54
	*Average*	*689.12*	*17.23*	*5.03*	*1835.05*
	*St. Dev.*	*9.22*	*0.23*	*1.04*	*55.73*

**Table 11:** Tensile test results for PLA+CF specimens—effect of lubricant exposure.

Case	Specimen Code	Max. Force [N]	Tensile Strength [MPa]	Fracture Strain [%]	Young’s Modulus [MPa]
*Case 12*	PLA+CF_6302H1	826.27	20.66	2.49	2240.74
	PLA+CF_6302H2	813.91	20.32	2.42	2199.22
	PLA+CF_6302H3	807.90	20.20	2.48	2205.80
	PLA+CF_6302H4	803.24	20.08	3.09	2198.08
	PLA+CF_6302H5	802.91	20.07	2.91	2186.36
	*Average*	*810.65*	*20.27*	*2.68*	*2206.04*
	*St. Dev.*	*9.64*	*0.24*	*0.30*	*20.62*
*Case 18*	PLA+CF_6302H71	741.26	18.53	2.23	1881.53
	PLA+CF_6302H72	757.41	18.94	4.21	1970.91
	PLA+CF_6302H73	776.43	19.41	3.10	1908.17
	PLA+CF_6302H74	803.04	20.07	6.05	1948.12
	PLA+CF_6302H75	815.08	20.38	3.89	1741.81
	*Average*	*778.64*	*19.47*	*3.90*	*1890.11*
	*St. Dev.*	*30.73*	*0.77*	*1.43*	*89.84*
*Case 19*	PLA+CF_6302H31	648.50	16.21	5.36	1762.53
	PLA+CF_6302H32	650.32	16.26	5.94	1821.66
	PLA+CF_6302H33	674.35	16.86	2.54	1779.45
	PLA+CF_6302H34	676.05	16.90	2.78	1726.89
	PLA+CF_6302H35	652.82	16.32	3.05	1784.96
	*Average*	*660.41*	*16.51*	*3.93*	*1775.10*
	*St. Dev.*	*13.60*	*0.34*	*1.59*	*34.52*

## Data Availability

Data sharing available upon request.
